# Deaths, injuries and detentions during civil demonstrations in Sudan: a secondary data analysis

**DOI:** 10.1186/s13031-019-0199-8

**Published:** 2019-05-02

**Authors:** Maysoon Dahab, Nada Abdelmagid, Ahmed Kodouda, Francesco Checchi

**Affiliations:** 1 Independent public health consultant, London, UK; 2 Faculty of Epidemiology and Population Health, London School of Hygiene and Tropical Medicine, Keppel St, London, UK; 3 Department of Political Science, Columbian College of Arts and Science, George Washington University, 2115 G St. NW, Washington, DC USA

**Keywords:** Deaths, Injuries, Mortality, Violence, Intentional, Demonstrations, Human rights, Sudan

## Abstract

**Background:**

Since December 2018, the latest wave of anti-government protests in Sudan has led to deaths, injuries and detentions. We estimated the number of people killed and described patterns of deaths, injuries and detentions up to 9 April 2019.

**Methods:**

We tabulated data from three publicly available lists maintained by Sudanese civil society sources (the Independent Movement, the Sudan Doctors’ Union and the “Lest We Forget” project), and applied to these a capture-recapture statistical technique that models the overlap among lists to estimate the number of deaths not on any list.

**Results:**

We estimated that about 117 civilians were killed in demonstrations during the above period, a considerably larger number than hitherto reported. Most decedents and injury victims were shot.

**Conclusions:**

This analysis demonstrates the importance of real-time data on political violence collected by civil society initiatives. The de facto Sudanese government should immediately cease attacks against peaceful civilian protesters and put in place guarantees for their safety.

**Electronic supplementary material:**

The online version of this article (10.1186/s13031-019-0199-8) contains supplementary material, which is available to authorized users.

## Background

The most recent wave of anti-government protests in Sudan began on December 19, 2018 in Atbara and spread to other major cities including the capital, Khartoum [1]. The demonstrations were initially against rising costs of living but quickly escalated into calls for the National Congress Party-led government to cede power. According to various reports, security agencies responded with force, including tear gas, rubber bullets and live ammunition [2]. At the time of writing, a de facto military government had assumed power.

Despite the previous government’s state of emergency declaration and banning of rallies, protests and civil disobedience continued, and reports of injuries, arrests and killings of unarmed civilians accumulated [3]. The former Government of Sudan confirmed that 29 people have died in the course of the protests [4], but other reports suggest the death toll is almost twice as high [5, 6]. There are also reports of hundreds of injuries and arrests in connection with the demonstrations [5–7].

At the time of writing, despite the lifting of a state of emergency protests continued country-wide. We sought to estimate the total number of civilians killed, and tabulate existing data on killings, injuries and detentions to better characterise the extent and typology of violence during the current wave of civil demonstrations in Sudan.

## Methods

### Data sources

We consulted three independent lists of people killed, injured or detained in the protests nationally, as of 9 April 2019. The lists were shared publicly on social media platforms and maintained by the Independent Movement [8] (until 21 February 2019; later data obtained from the organisation's public email, info@almustagleen.org), the Sudan Doctors’ Union [9, 10] and an anonymous Google Site (“Lest We Forget”) [11] (note that the dataset uploaded at the time of writing by "Lest We Forget" contains fewer observations than when we accessed it as of 28 February 2019; no explanation was provided for this difference). Information in Arabic was translated by the authors into English.

The Independent Movement, a Sudanese diaspora-led organisation [8], compiles and publishes the names of those killed or detained in Sudan since the 5 January 2018. The names are obtained from open sources, primarily social media platforms, and verified through a network of ground informants established in November 2017. Informants verify the names and incidents with the relatives, friends or political party affiliates of those killed or detained. The list is updated on a weekly basis.

The second list is maintained by the Sudan Doctors’ Union, a union revived in 2012 by doctors who were dissatisfied with the state of the public health system [7]. The Union’s Central Committee of Sudan Doctors (CCSD) coordinates medical care to injured protesters [12] and documents deaths and injuries associated with demonstrations, compiled through first-hand notifications by Union members working in health facilities across Sudan, as well as through open sources (largely social media platforms). The CCSD verifies information through its network of doctors throughout the country. The names of those killed and injured are regularly shared through the CCSD’s social media pages.

Finally, “Lest We Forget” is an anonymous website [13], created on 23 December 2018. The list compiles the names of those killed from social media platforms and cross-checks them from multiple open sources.

### Analysis

**Fig. 1:**
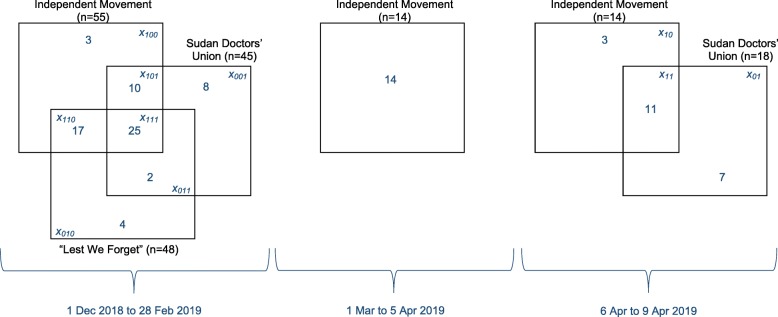
Availability of and overlap among the three lists of deceased people used for this analysis

To estimate total killings, we manually cleaned and removed duplicates from all three lists and linked the records across lists based on name of the deceased, gender, date, place and cause of death, and site of injury for those shot dead. We excluded deaths of army or security personnel from the analysis and two unidentified decedents with insufficient details. As shown in Fig. 1, while all three lists were available up to end February 2019, the Independent Movement was the sole source available to us up to 5 April, with the Doctors’ Union also reporting during 6–9 April when a protest sit-in and related violence unfolded in central Khartoum.

For the period up to end February, we used three-list capture-recapture analysis [14] (multiple systems estimation) with Bayesian Model Averaging to estimate the true number of deaths. Briefly, this technique rests on fitting statistical models to the observed overlap among lists: model coefficients supply predicted values for each *x*_*ijk*_ category, where *i*, *j* and *k* are 0–1 booleans that denote whether a death occurs in list *i* (Independent Movement), *j* (“Lest We Forget”) and *k* (Sudan Doctors’ Union), whereas *x* is the number of deaths in that *i,j,k* category (Fig. 1). We fit Bayesian log-linear Poisson models with uninformative priors to the contingency table formed by each possible *x*_*ijk*_ category. IndependentEight possible models can be fit depending on which interactions among lists are assumed to exist (e.g. whether being on listed by *i* alters the probability of being listed in *j*, etc.) [15]. The predictions of each candidate model can be used to compute *m*_*000*_, i.e. the estimated *x*_*000*_ (deaths that do not appear on any list), as

$$ {m}_{000}=\frac{m_{111}{m}_{100}{m}_{010}{m}_{001}}{m_{110}{m}_{101}{m}_{011}} $$

, with 95% confidence intervals (95% CIs) provided by the 2.5th and 97.5th percentile of the likelihood profile.

We computed the average *m*_*000*_ from all models as ${m}_{000}={\sum}_{i=1}^{i=K}{m}_{000,i}{\mathit{\Pr}}_i$ where K is the total number of models averaged over, i is one of these models and Pr is the posterior probability of that model. We summed *m*_*000*_ to deaths appearing on at least one list to compute the total number of deaths. An annotated R statistical code is included as Additional file 1. This general approach has been extensively used in human rights analysis to date, e.g. for estimation of killings during the wars in Kosovo [16] and Timor Leste [17].

We summed to the above deaths the total reported by the Independent Movement up to 5 April, the latter sub-period total inflated to reflect the sensitivity (i.e. completeness) of that source as estimated up to 5 April (see Results). For 6–9 April we used simple two-list capture-recapture estimation [14], whereby ${m}_{00}=\frac{x_{10}{x}_{01}}{x_{11}}$, and added the resulting sub-period estimate to the overall total.

For injuries and detentions, we merely compiled data available from the Sudan Doctors’ Union and the Independent Movement respectively, and tabulated key variables.

## Results

Overall, the three lists contained unique records for 104 decedents, with moderate overlap among them (Fig. 1). The Table 1 shows the eight candidate models fitted to the three-list data up to end February 2019, and the resulting Bayesian Model Average, suggesting some 6 (95%CI 2–31) deaths were not captured by any list. This estimate, added to the 69 deaths on any list, yields a total estimated death toll of 75 (95% CI 71–100) up to 28 February 2019. Accordingly, the estimated sensitivity of individual lists during this period was 73% (95% CI 55–78%) for the Independent Movement, 64% (95% CI 48–68%) for the “Lest We Forget” project and 60% (95% CI 45–63%) for the Sudan Doctors’ Union, while all three lists together captured some 92% (95%CI 69–97%) of all deaths.

**Table 1:** Estimated number of people killed according to each candidate log-linear model and overall, based on Bayesian model averaging (Dec 2018-Feb 2019)

Model (interaction terms included)	Degrees of freedom	Bayesian Information Criterion	Estimate of deaths not on any list or *m*_*000*_ (95% CI)	Posterior probability
1. No interactions	3	0.30	2 (1–4)	0.036
2. [Independent Movement x “Lest We Forget”]	2	−5.07	5 (2–12)	0.531
3. [Independent Movement x Sudan Doctors’ Union]	2	4.53	2 (1–5)	0.004
4. [“Lest We Forget” x Sudan Doctors’ Union]	2	0.30	1 (0–3)	0.036
5. [Independent Movement x “Lest We Forget”] + [Independent Movement x Sudan Doctors’ Union]	1	−2.87	16 (3–127)	0.177
6. [Independent Movement x “Lest We Forget”] + [“Lest We Forget” x Sudan Doctors’ Union]	1	−2.77	2 (0–9)	0.168
7. [Independent Movement x Sudan Doctors’ Union] + [“Lest We Forget” x Sudan Doctors’ Union]	1	4.45	1 (0–3)	0.005
8. [Independent Movement x “Lest We Forget”] + [Independent Movement x Sudan Doctors’ Union] + [“Lest We Forget” x Sudan Doctors’ Union] (saturated)	0	0	7 (1–81)	0.042
Bayesian model average			6 (2–31)	1.000

During 1 March to 5 April 2019, a further 14 deaths were reported by the Independent Movement, while during 6–9 April two-list capture-recapture analysis suggested 2 unlisted deaths occurred alongside 21 appearing on either or both available lists (Fig. 1). Accordingly, the death toll over the entire period is likely to be at least 112 (75 + 14 + 23), or about 117 (75 + 14/0.73 + 23) if we assume the estimated sensitivity of the Independent Movement source remained constant up to 5 April, and adjust for this.

Of the 104 decedents reported by at least one list, 4 (4%) were female and 100 (96%) male, while at least 15 (14%) were aged below 18 years. Of 89 with a reported cause of death, 66 (74%) were killed by gunshot (10 in the head, eight in the chest, five in the abdomen, one in the neck, one in the hip and the remainder in an unspecified site); eight killed by an explosive device; six tortured while in detention; five suffocated by tear gas; and four run over by security vehicles. At least two medical professionals were reported shot while at work in hospitals.

The deadliest day of the protests was 20 December 2018 with 34 people killed, 25 in Al-Gadarif alone, the latter being the city with the most killings (30/104 or 29%) after Khartoum State (55/104 or 53%). The Sudan Doctors’ Union provided information on injuries gathered from hospitals in Al-Gadarif between 19 December 2018 to 1 March 2019. Of the 24 individuals reported injured, 22 (96%) were male. Among the 14 for whom the cause of injury was reported, all but one (a policeman injured by an exploding tear gas canister) were shot, seven (53%) in the legs or thighs, two in the abdomen, two in the head, two in the chest and one in the shoulder.

Data on people detained during demonstrations were published by the Independent Movement up until 8 April 2019. Of 934 individuals listed in this database, 295 (32%) were described as activists, 188 (21%) as politicians, 79 (9%) students, 68 (7%) medical professionals, 64 (8%) lawyers, 61 (7%) journalists and 53 (6%) teachers. There were 151 female detainees (19%) and at least 6 minors.

## Discussion

Our analysis suggests that a considerably larger than hitherto reported number of civilians were killed during protests in Sudan, and that the different civil society-maintained lists of decedents, while providing a precious record of time, person and place patterns, should be analysed jointly in order to estimate a credible total.

Though rapid and based on fragmentary data, our analysis also points to a consistent pattern of deaths and injuries by firearm. However, other causes of mortality, notably torture, were also reported. These data corroborate existing evidence on intent to kill or harm by government-directed security forces, and suggest countrywide, sustained use of live ammunition against protesters. A large number of protesters appear to have been detained, with professionals (including doctors) making up a significant proportion. We found no data on the current status of these detainees, but others have reported systematic torture and abuse at detention facilities [18].

These patterns of killings, detention and torture are similar to previous incidents of popular protests in Sudan and elsewhere. During anti-austerity protests in 2013, the Government of Sudan reported an official death toll of 85, while Sudanese civil society organisations reported higher numbers, ranging from 170 to 230; most of those killed were shot while some died in detention [19, 20]. Regionally, governments have employed similar tactics in response to popular dissent. For example, during the January 2011 Egyptian uprising, some 840 people were killed, with reports of many protesters dying as a result of shots fired to the upper body, including the head or chest, and security forces driving into protesters in armoured vehicles [21]. Demonstrations that began over social security reforms in Nicaragua in 2018 also met with excessive use of force and arbitrary detentions, including reports of torture and gunshot wounds [22]. While the government acknowledged 195 deaths as of 3 August 2018, other independent estimates varied from 317 to 450 [23]. A number of deaths amongst protesters were not reported to official authorities due to fear of reprisal or mistrust [24].

### Limitations

Paucity of data describing individual deaths or injuries prevented us from fully characterising the circumstances of these events, inferring intentionality or attributing responsibility. Capture-recapture analysis is an established method, but it is possible that none of the lists had sufficient on-the-ground coverage to detect deaths in smaller towns or outlying regions of Sudan. The technique relies strongly on the accuracy of record linkage (we encountered no instances of equivocal records, e.g. similar names). It also assumes independence among lists, which in this analysis cannot be fully established, as two of the lists (the Sudan Doctors’ Union and “Lest We Forget” project) both consulted social media, the former not exclusively; however, at least for the period up to 28 February 2019, our three-list analysis explicitly allows for non-independence assumptions to be relaxed by including models with between-lists interaction terms. We could only apply this recommended capture-recapture technique over a fraction of the analysis period, possibly resulting in under- or over-estimation of the period-wide death toll. Moreover, additional deaths among detained protesters may have occurred that data sources were not (yet) aware of, resulting in under-estimation. Lastly, our description of patterns in injuries and detentions relies on a single source, which may not have captured the true totals: these patterns should therefore be treated with some caution.

## Conclusions

This analysis of existing secondary data confirms that lethal force has been used against civilian demonstrators in Sudan, and suggests that a substantially larger number of people may have been killed than has so far been reported. The de facto Sudanese government should immediately cease attacks against peaceful civilian protesters and guarantee their safety across Sudan, while releasing all remaining detainees and enabling access to free healthcare for any injured protesters.

Civil society sources of data on killings and other events are essential to produce adequate documentation of potential crimes in such situations: these data collection initiatives should continue and be supported. Applying statistical analysis to such data is likely to become a more frequently available option for rapid or real-time estimation of political violence, as such data become increasingly available, e.g. through social media. Such analysis can illuminate the extent of under-estimation in death tolls reported widely by government, international non-governmental organisations and media sources, and establish more credible figures.

## Additional file

Additional file 1: Authors' R statistical analysis code for three-list capture-recapture analysis. (R 17 kb)

## Abbreviation

CCSD: Central Committee of Sudan Doctors

## References

[1] Sudan price protests subverted by 'infiltrators': spokesman [https://www.reuters.com/article/us-sudan-protests/eight-killed-as-price-protests-spread-in-sudan-idUSKCN1OJ0XT].

[2] Dozens have been killed by the regime. But Sudan’s protesters march on [https://www.theguardian.com/world/2018/dec/30/dozens-have-died-but-sudan-protesters-march-on].

[3] Sudan's Al-Bashir Declares State of Emergency for One Year [https://www.bloomberg.com/news/articles/2019-02-22/sudan-s-al-bashir-declares-state-of-emergency-for-one-year].

[4] Sudan protests death toll rises to 29 - investigatory committee [https://uk.reuters.com/article/uk-sudan-protests-casualties/sudan-protests-death-toll-rises-to-29-investigatory-committee-idUKKCN1PI2RX].

[5] Reports of excessive force against Sudan protests deeply worrying – Bachelet [https://www.ohchr.org/en/NewsEvents/Pages/DisplayNews.aspx?NewsID=24080&LangID=E].

[6] Seven things you should know about the unrest in Sudan [https://www.amnesty.org/en/latest/campaigns/2019/01/seven-things-you-should-know-about-the-unrest-in-sudan/].

[7] How an illegal Sudanese union became the biggest threat to Omar Al Bashir’s 29-year reign [https://www.thenational.ae/world/africa/how-an-illegal-sudanese-union-became-the-biggest-threat-to-omar-al-bashir-s-29-year-reign-1.819159].

[8] List of people killed or arrested by Albashir’s militias between 13th December 2018 and and 21st February 2019 in Sudan [https://pyschotec.com/wp-content/uploads/2019/02/List-of-people-shot-dead-or-arrested-in-Sudan-190221.pdf].

[9] Sudan Doctors' Committee Facebook page, post from 19 March 2019[https://m.facebook.com/story.php? story_fbid=2285845735034663&id=1775598556059386.

[10] Sudan Doctors' Union Facebook page, post from 9 April 2019. [https://www.facebook.com/sudandoctorunion/posts/2630513253657154.

[11] List of martyrs in Sudanese cities [https://docs.google.com/spreadsheets/d/1qS4mxewKKE7AlNUS6b38DbRlXDg4KPutBtSY5e5GI/edit#gid=174144963].

[12] Statement delivered by Mr A Hassan, Senior Clinical Fellow in Neurosurgery and Vice Secretary General of Sudan Doctors' Union UK [https://cdn-cms.f-static.com/uploads/905961/normal_5c6dc83a76036.pdf].

[13] Lest We Forget [https://sites.google.com/view/Sudan-revolution-19-dec].

[14] International Working Group for Disease Monitoring and Forecasting (1995). Capture-recapture and multiple-record systems estimation I: history and theoretical development. Am J Epidemiol.

[15] Fienberg SE, Johnson MS, Junker BW (1999). Classical multilevel and Bayesian approaches to population size estimation using multiple lists. J R Stat Soc A Stat Soc.

[16] Ball P, Betts W, Scheuren F, Dudukovich J, Asher J (2002). Killings and refugee flow in Kosovo, march-June 1999: a report to the international criminal Tribunal for the Former Yugoslavia. Book Killings and Refugee Flow in Kosovo, March-June 1999: a Report to the International Criminal Tribunal for the Former Yugoslavia.

[17] Silva R, Ball P (2006). The profile of human rights violations in Timor-Leste, 1974–1999: a report by the Benetech human rights data analysis group to the commission on reception, truth and reconciliation of Timor-Leste. In *Book The Profile of Human Rights Violations in Timor-Leste, 1974-1999: A Report by the Benetech Human Rights Data Analysis Group to the Commission on Reception, Truth and Reconciliation of Timor-Leste* (Editor ed.^eds.).

[18] What happens inside Sudan’s secret detention centres? [https://www.bbc.co.uk/news/av/world-africa-47216487/what-happens-inside-sudan-s-secret-detention-centres].

[19] Excessive and deadly: the use of force, arbitrary detention and torture against protesters in Sudan [https://www.amnesty.org/download/Documents/4000/afr540202014en.pdf].

[20] Over 170 dead, including 15 children, and 800 detained as demonstrations spread throughout Sudan [http://www.acjps.org/wp-content/uploads/2013/10/4-10-13-Over-170-dead-including-15-children-and-800-detained-as-demonstrations-spread-throughout-Sudan.pdf].

[21] Egypt rises: Killings, detentions and torture in the '25 January Revolution**'** [https://www.amnesty.org/download/Documents/32000/mde120272011en.pdf].

[22] Gross Human Rights Violations in the Context of Social Protests in Nicaragua [http://www.oas.org/en/iachr/reports/pdfs/Nicaragua2018-en.pdf].

[23] OAS human rights body counts 317 dead in Nicaragua unrest [https://www.apnews.com/3cde1a64e59a4c8ea1d472f144aedd6b].

[24] Instilling Terror: from lethal force to persecution in Nicaragua [https://www.amnesty.org/download/Documents/AMR4392132018ENGLISH.PDF].

